# "The Hospital" Nursing Mirror

**Published:** 1897-05-01

**Authors:** 


### "The Hospital" Nursing Mirror.

Being the Nursing Section of "The Hospital."

[Contributions for this Section of "The Hospital" should be addressed to the Editor, The Hospital, 28 & 29, Southampton Street, Strand, London, W.C., and should have the word "Nursing" plainly written in left-hand top corner of the envelope.]

### News from the Nursing World.

### THE DUCHESS OF YORK.

The news that a little daughter has been born to the Duke and Duchess of York has been received with general satisfaction. On Saturday last alarming rumours as to the Duchess's health were current, and it was with a feeling of much relief that the reassuring news from Sandringham has been read. On Sunday the following bulletin was issued by Sir John Williams and Dr. Manby: "York Cottage, Sandringham, Sunday. Her Royal Highness the Duchess of York gave birth to a daughter at half-past three this afternoon. The Duchess and her infant daughter are well." The latest addition to the Royal Family brings the number of the Queen's grandchildren up to thirty.

### A HOSPITAL WEDDING.

There was a large gathering at Bath Abbey on Wednesday, the 22nd ult., when Miss Florence Sellars, lady superintendent of the Lansdowne Grove Hospital, Bath, was married to Mr. Frederick Bathurst, son of the late Colonel Bathurst, of the Coldstream Guards. The bride was attended by four nurses in uniform, and was given away by Dr. Percy Wilde, physician to the hospital. Miss Sellars was presented with an illuminated address by the committee, stating that, entering the hospital as probationer in 1888, she had passed through the ranks of staff nurse and sister, being finally appointed lady superintendent, during the whole of which time her brightness, unselfishness, and ready sympathy had endeared her to all. Many of the poor patients assembled at the Abbey to bid good-bye to "Sister Florence." Miss Sellars was presented with several handsome presents, including a purse of £55 from the Bath Ladies' Work Society, a diamond ring from the medical staff, and a silver fish service from the nursing staff. After the wedding a reception was given by the medical staff of the hospital.

### EAST-END MOTHERS' HOME.

The annual meeting of the East-end Mothers' Home was held recently at the Home in Commercial Road. The Rev. Marmaduke Hare, who presided, said that the past year had been one full of work. There had been more women in the Home than ever before, more children had been born, and the mortality had been less. He spoke earnestly of the immense importance in the East-end of London of such an institution, where not only did the poor mothers receive every care and attention during the time of childbirth, but where they also learnt necessary truths respecting cleanliness and the management of infants, which remained with them long after they left the Home. The income last year met the expenditure, but there is still a debt of £1,200 on the building. A special fund has been opened for the purpose of clearing off this liability, to which liberal donations are urgently needed; but the committee trust the general funds will not be allowed to suffer, as the ordinary income is barely sufficient to meet the daily needs of the work. Funds are much required, not only to keep up the present work, but to extend it. At the meeting a vote of thanks was passed to the medical staff and honorary officers, and to Miss Blomfield, the lady superintendent.

### "THE CHILDREN'S TRIBUTE."

"The Children's Story of the Queen's Reign," in connection with the "Children's Salon," is being told in many parts of the country on behalf of the Queen's Jubilee Institute for Nurses. Meetings have taken place this week in Chester, Manchester (where the Lady Mayoress, Miss Foulkes-Roberts, is interesting herself much in the movement), and Bradford. The lecture will be given in the Egyptian Hall of the Mansion House, the Lady Mayoress in the chair, on May 7th.

### "MOY-MELL."

On May 3rd a grand fête in aid of the Children's Hospital, Temple Street, Dublin, is to be inaugurated in the Rotunda Buildings and grounds. "Moy-Mell" is being held under very influential patronage, and the Lord Lieutenant and Lady Cadogan are to open the proceedings on the 3rd. The Children's Hospital does an excellent work, and is the oldest institution of its kind in Ireland. We hope the fête will prove a thorough success, and that the efforts of those who are working so hard in the cause of the hospital will result in a satisfactory addition to its funds.

### CANADA AND THE QUEEN'S COMMEMORATION.

The establishment of a "Victorian Order" of nurses in Canada in commemoration of the sixtieth year of the Queen's reign is rapidly taking shape. The Countess of Aberdeen is presiding over the movement, and taking the deepest personal interest in its formation, whilst it has also the patronage of the Governor-General, the Lieutenant-Governors of the provinces, Sir Mackenzie Bowell, and many distinguished people. The urgent need for trained nurses and the necessity for improving the conditions under which these trained nurses carry on their labours, is well set out in an announcement issued by the provisional committee. The scheme aims at helping the hospitals to train more women for the work, and securing the immediate services of skilled nurses for the poorer members of the population. Local committees are to be formed all over Canada, and subscription funds opened, it being hoped that in this way a sum of one million dollars, sufficient to endow the order permanently, will eventually be raised. It is proposed when the order is formed that the members may be—(*a*) Nurses who are already graduates in schools of recognised standing, and who pass an examination such as may be prescribed. (*b*) Nurses who shall be specially trained for the order and who shall pass the prescribed examination. The qualifications of the nurses of the Victorian Order are to be of the highest for the class of work they are expected to do. Some of the chief objects of the order will be:

(*a*) To provide skilled nurses in sparsely settled and outlying country districts; (*b*) to provide skilled nurses to attend the sick poor in their own homes in cities; (*c*) to provide skilled nurses to attend cases in cities at fixed charges for persons of small incomes, the charges being paid to the funds of the order; (*d*) to provide small lying-in rooms or wards in cottage hospitals or homes; (*e*) to prepare trained nurses thoroughly qualified to carry out these objects.

### ENGLISH NURSES FOR THE EAST.

Six more nurses left England on Wednesday for Athens. They are Miss Florence Sherman and Miss Isabella Coombes, from Guy's Hospital; Miss H. E. Nisbet, King's College Hospital; Miss Gertrude Johnson and Miss Henrietta Whiteford, of the Nurses' Co-operation; and Miss Clara Hill, of the Registered Nurses' Society. This brings the total number of English nurses sent out to Greece to 19. The Crown Princess of Greece, at whose request the nurses have gone out, has guaranteed their travelling expenses, and the salaries are to be defrayed by the Grecian Nursing Committee. Private sympathisers have contributed largely to the Fund being raised, and at a meeting this week it was resolved to relieve the Crown Princess of any expense in the matter of equipping and sending out the nurses. The following ladies are on the Committee (of which Mrs. Bedford Fenwick is the Hon. Secretary):—Mrs. Gladstone, Dowager Countess Russell, Lady Henry Somerset, Lady Stevenson, Lady Joicey, Lady Dilke, Lady Osborne Morgan, Mrs. Asquith, Mrs. Wickham, Mrs. Labouchere, Mrs. F. S. Stevenson, Mrs. Craigie, and Mrs. John Burns. The nurses who have already reached Greece met with an enthusiastic reception. Miss Monk (King's College Hospital) has received an interesting letter, dated Athens, April 21st, from Miss Alice Davies, one of her nurses, who left England with three other volunteers on Good Friday. Miss Davies tells of the welcome of the party by the Crown Princess, at whose palace they were received on their arrival. They expected to proceed next day to the front on ambulance duty.

### THE DERBYSHIRE ROYAL INFIRMARY.

Miss Carvosso, matron of the Derbyshire Royal Infirmary, has resigned her post on account of ill-health. At the last half-yearly meeting of the institution, a resolution was passed to the effect that "Miss Carvosso's resignation be accepted with extreme regret, and that in accepting her resignation in consequence of ill-health the Board desire to place on record their high appreciation of the services which as matron she has rendered to the infirmary during the past four years. The Board desire to bear special testimony to Miss Carvosso's services during the exceptional period of rebuilding and refurnishing. The duties of matron during this period have been onerous in the extreme, and Miss Carvosso has devoted all her energy at the sacrifice of health to the interests of the institution."

### TWO ROYAL NURSES.

The fame of Duke Charles Theodore of Bavaria as an oculist is widespread, but it is perhaps not so well known how ably he is seconded in this beneficent work by his wife and daughter, both ladies taking the greatest interest in the Duke's patients, and devoting much of their time to nursing. The Duke has three ophthalmic hospitals, one being in the palace at Tegernsee, the others at Munich and Meran. The Duchess Maria Josepha and her daughter when helping the Duke at his operations wear black uniform dresses with linen collars and cuffs, and large aprons.

### A NURSING FUND FOR ST. IVES.

Endeavours are being made in St. Ives to place on a permanent footing the Nursing Fund started a few years since in that town by the late Mrs. Leslie Stephen. At her initiation, in 1892, a committee was formed, and sufficient donations and annual subscriptions raised to enable a trained nurse to be engaged to work amongst the sick poor. The nurse's labours have met with great appreciation. The scheme has been carried on at a yearly cost of about £70, of which £50 has come from annual subscriptions, the remainder being made up by donations and by the interest on a small invested sum. A permanent endowment is now suggested as an appropriate memorial to Mrs. Leslie Stephen, and an appeal is being made to those friends "who cherish her memory, and who are aware of the strong interest she took in all movements of the kind, and of her affection for St. Ives." Miss Duckworth, Mrs. Stephen's daughter (who is president of the Nursing Association), with other members of her family, are intending to contribute to this fund, which it is proposed to call, after its originator, the "Julia Prinsep Stephen Nursing Fund."

### ROTUNDA HOSPITAL, DUBLIN.

On Monday, April 26th, Lady Roberts, wife of Field-Marshal Lord Roberts, Commander of the Forces in Ireland, performed the ceremony of naming the wards of the new gynæcological wing of the Rotunda Hospital, Dublin. The governors issued invitations for a large party on the occasion. The wards have been called respectively the Iveagh, the Thomas Plunket Cairns, and the Eleanor and William J. Smyly, the latter being named after the ex-master of the hospital and his wife, who worked arduously for the good of the hospital, and helped much towards the erection of the auxiliary wing.

### SHORT ITEMS.

The well-known firm of Marcus Ward and Co. is publishing a photogravure depicting the Queen as she appeared at her coronation and as at the present day, in commemoration of the 60th year of Her Majesty's reign. The profit realised by the sale of the picture is to be handed over to the Queen's Jubilee Institute for Nurses.—It has been decided to build a cottage at Hucknall for the accommodation of the district nurses, by way of commemorating the present year. The Duke of Portland has been asked to give a site for the purpose.—A sister of the German Emperor, the Princess Frederick Leopold, has undertaken a course of practical instruction in sick nursing under Dr. Paunwitz, of the Imperial Board of Health.—Mr. Bancroft has promised to give his reading of Dicken's "Christmas Carol" in aid of the Colonial Nursing Association on June 3rd, at the Imperial Institute.—A detachment of the Russian Red Cross Society left St. Petersburg on April 27th for the seat of the Turco-Greek war. This consists of two doctors and twenty Sisters of Mercy, under the direction of a surgeon, and the party are taking with them all the accessories for a hospital of 50 beds.

### Lectures to Surgical Nurses.

By H. A. Latimer, M.D. (Dunelm), M.R.C.S. of Eng., L.S.A. of London, Consulting Surgeon, Swansea Hospital; President of the Swansea Medical Society; Lecturer and Examiner of the St. John Ambulance Association, &c.

### VI.—RESULTS OF HÆMORRHAGE—SYMPTOMS AND TREATMENT OF FAINTING AND COLLAPSE—REACTION: ITS SYMPTOMS AND TREATMENT.

You will have gathered from what I told you in the preceding lecture that in modern surgery much care is exercised to limit the loss of blood during and after operations. Surgeons are no longer lavish in the waste of this precious fluid. There was a time, not so far distant either, when people were bled for the cure of all sorts of disorders; and blood was "let" often to ward off imaginary evils which it was feared might occur, many persons being bled as a routine measure at the spring of the year, with the idea that by such a proceeding they would be kept in good health. Now, we know that the loss of a large quantity of blood is severely felt by its owner, and that although its quantity is soon replaced in the vessels after hæmorrhage has occurred, its quality is but slowly repaired. Hence, it results that much attention is paid to the prevention and control of bleeding, and that it behoves you to know what follows on excessive bleeding, and what is to be done by you in nursing patients in whom the same has occurred.

The immediate result of much blood being lost is "syncope," or fainting. This symptom owes its cause to an insufficient supply of blood reaching the brain, when this organ does its work so feebly that general loss of consciousness takes place. You may know when a person is fainting by his pale face; if the condition is complete and he absolutely loses consciousness, he falls to the ground, his muscles being completely relaxed, and whilst lying there his pupils are dilated, and hardly any pulse is to be felt at the wrist. As a rule, a fainting person soon recovers on being treated appropriately. The immediate cause of the condition being the want of a sufficient supply of blood to the brain, it stands to reason that the lower the head is placed the more easily blood will flow to the brain; therefore, let this be always present in your mind, and remember that a fainting person should be laid flat, and with the head, if anything, lower than the body. Then you may fan him to rouse him, and some stimulant, like smelling salts, may be applied to the nose. If the fainting condition continues for some time, we then have a state which is known as that of "collapse," or shock, which is no more or less than a more serious condition of fainting, and one which generally owes its origin to some more serious cause than the former. Note that in both these conditions of fainting and collapse you have to deal with a heart which is acting feebly. Both these conditions often arise from causes other than hæmorrhage. The dread of an operation, a fright, some sudden alarm, or a severe pain, cause them: but in all cases the treatment of the condition is alike. Fainting you must have often seen in the wards of the hospital, and probably you have often seen conditions of collapse or shock. I will now detail one such case as a type of shock, premising that shock is the message sent to the brain, and that collapse of the system is the response to that message. I was called not long ago to see a man who had suddenly vomited a large quantity of blood from the stomach; when I came to him I found him pale in the face, sweating, semi-conscious, with so feeble an action of the heart that I could hardly count his pulse; during the general muscular relaxation which had taken place his bowels had acted. Here is a typical example of a state of shock. The treatment I carried on is the one which I shall now detail to you, as being the appropriate one for that state; but I call you to notice particularly that collapse is only continued fainting—that it is distinguished from the latter state only by the fact that there is no sudden and rapid recovery from it, as occurs in the milder condition. Your treatment then, will have to be longer continued than what it would be in fainting, as you have to deal with a state which is far more serious. You will notice that the person will be cold, and that if you take the temperature with a thermometer it will show a sub-normal condition; therefore this supplies you with an indication. You will keep him warm by applying hot bottles to the feet and legs, and covering him well with bed-clothes. Watch him also to see that he is kept lying down, for in this condition of collapse patients are very restless and are very apt to rise to get up, and they may often lose their lives in consequence of such a proceeding, the over-taxed heart failing altogether.

Under the direction of the medical man in attendance you will administer foods and stimulants, and amongst the best of the latter kind of agents you will find Liebig's extract of meat, given in large doses with hot water, act very well. Sometimes the condition is so profound, and the danger is so immediate, that the surgeons have to adopt the promptest measures for saving life. If it results from loss of blood one such measure is the operation of transfusion of blood. This is performed by the surgeon opening a vein in the arm of somebody who is willing to be a subject, and another vein in the arm of the collapsed person, and the blood is passed, with a suitable instrument, from the one person to the other; or the blood given by the sound person is received in a vessel, whipped round to deprive it of its fibrin, filtered, and the filtered blood is injected into the vein of the sufferer. It has been found of late years, however, that the injection of some saline such as common salt, dissolved in water, into the bowel, exercises a prompt and restorative effect, and this measure is more often adopted than the one of transfusion. Another means adopted to resuscitate a collapsed person is by the subcutaneous injection of ether. These measures proving successful, the patient now runs a risk of passing into the opposite condition known as "re-action." During the fainting and collapsed state the brain received an insufficient amount of blood, and the heart acted feebly; now the reverse obtains. The face becomes flushed, the pulse is quickened, the breathing becomes more rapid, the temperature of the body is apt to be raised, and, instead of having a state of mental stupor, the patient becomes excited. In the old days this stage was very much feared, was looked out for, and was often dealt with by measures for lowering the patient; now nothing of the sort is done; its treatment is by maintaining a condition of quiet, by reassuring the sufferer, and by continuing to nourish him with proper foods, of an easily digestible kind. It is, as a rule, not a condition which prevails for long; it is the other swing of the pendulum, and, the causes which have set it in motion subsiding, all goes well.

Passing on from these accompaniments of disease, we will turn to the consideration of burns and scalds. You are all familiar with both these states, as you see them often. Burns are the injuries caused by the application of dry heat, scalds those resulting from moist heat. In considering these injuries it is not necessary to go into very great refinements, it will be sufficient for you to remember that they may present themselves in three different degrees; first, there is that of mere transient redness of the part; secondly, there is the formation of blisters—vesication; thirdly, there is actual death of a portion of the true skin, and may be of parts beneath. These must be considered separately.

### Post-Graduate Clinics for Nurses.

By a Trained Nurse.

### XI.—THE DOMESTIC ECONOMY OF ILLNESS.

In my little sermon of last week on some points with regard to the health of the private nurse there was no space wherein to draw attention to the important fact that the physical condition of the nurse assuredly reacts on her patient. This subject so far has hardly been discussed, but it is only logical to infer that contact with persons over-tired, with nervous systems out of order, may have a bad effect upon us when we are ourselves well, though we may be able to resist the influence, as we can, for instance, throw off the germ of typhus or scarlet fever. When ill, however, our sensitiveness in this direction is much increased, and it is undoubtedly injurious to the sick to be surrounded by tired, exhausted persons. A healthy nurse, on the other hand, is as invigorating to her patient as is a supply of fresh air. It seems reasonable to believe that good health is infectious—and most of us have experienced the feeling that the society of some vigorous persons seems as restorative as a visit to a bracing district. Patients have frequently told me they can tell by the touch of the hands whether the nurse attending them is tired or fresh—the light, breezy touch of the fresh nurse is cheering, the heavy languidness with which the exhausted nurse cares for her patient is distinctly depressing. The nurse, therefore, who would live her working life comfortably to herself, and exert a healing, brightening effect upon her patients, must needs pay attention to her health.

In the special courses for special nurses which I have suggested as my ideal for the future, some place must certainly be found for practical classes on the domestic economy of illness, a term which may need some explanation. As a first proposition I must lay down the axiom—which few will dispute—that the modern woman is losing, partially at least, the housewifely instincts of bygone times, and that the average modern girl has very hazy notions of practical housekeeping. And this lack of housekeeperliness is as grave a defect in a nurse as it is in any other woman, working or otherwise. In the repertoire of the private nurse, especially, domestic economy is a most valuable and treasured possession. The reasons prompting me to recommend lectures and practical demonstrations on the domestic economy aspect of the sick-room are manifold, the necessity for such practical teaching having forced itself insistently on my consciousness for several years past.

All of us are familiar with the proverbial argument brought against the employment of qualified nurses. "I cannot afford a trained nurse, it is so expensive," is the objection with which in modest households the doctor is frequently confronted, when the gravity of an illness suggests that its care should be consigned to skilful hands.

Since I acted for some time as Charge Sister of a private paying Hospital in London, and since it has been added to my province to be brought closely into contact with private nurses in their work, I have begun to understand this argument and believe I can trace the origin of the conviction that "trained nurses are so expensive." Investigation shows this conviction to be by no means based on the weekly salary given to a nurse. It is the hundred and one requirements which the employment of a trained nurse suggest which makes an illness in the house so expensive and dreaded a matter. For it must be acknowledged some private nurses, without a due regard for family finances, insist on the purchase of expensive luxuries and appliances for the sick-room, which are often used only once or twice. Now, a little ingenuity might have suggested an economical substitute for these articles of invalidism, an ingenuity on the part of their nurses which would be much appreciated in households whose income is moderate and to whose small means illness is an unwelcome strain. My experiences in the private hospital already mentioned gave me an insight into the expense which a want of domestic knowledge on the part of nurses might entail on their patient's friends; and it taught me also what an important bearing thrift and domestic economy might have on the curriculum of the Training School. Though some years have elapsed since this experience, time has not dimmed the memory of kettles of boiling water placed on decorative hearth-tiles, which were, in logical sequence, cracked. In two months two kettles were "boiled out" and in holes.

"Oh, Sister, I quite forgot the kettle; I am so sorry!" said a new probationer. And I vainly try to dismiss from my mind's eye the looks of irate housemaids coming with complaints and anathemas that "it was no use brightening the fire-irons and fenders, for the nurses were always rusting them with hot water." Spots of candle-grease and tell-tale patches of medicines, liniments, and ointments did not add to the beauty of the expensive carpets on the floors of that pretty Home; but all these things explained the rooted prejudice and feuds so often evinced by domestic servants against the presence in a household of trained nurses.

Twice during my brief tenure of office did I send for a plumber to remedy the complete stoppage of drains through an accumulation of match-sticks, pledgets of cotton-wool, bits of strapping and lint which had been thrown down heedlessly!

Full many a time and oft have I seen trained and certificated nurses, after completing a surgical dressing, empty away with the lotion lengths of bandage, "sponges" of wood-wool and gauzes which, to say the least of it, are effectual stumbling-blocks in the interior of drain pipes. Compared with these surgical impediments, the hair combings habitually thrown by housemaids into house-drainage are harmless and innocent. A Borough Surveyor once said to me: "Trained nurses by their talent for stopping drains cause even more trouble than do domestic servants." Now, this is a terrible indictment. But each one of us looking back on the days when we were young and in the harness of hospital training will recall without difficulty the manifest impossibility of any drain-pipe devised by science carrying away the heterogeneous substances thrown down not only by new "pros." and trained nurses, but—humiliating though the disclosure may be—even by Sisters doing active duty in busy times and "taking-in" seasons.

Some elementary and practical sanitation then should form a part of the curriculum of a nurse's education. Hygiene—of a sort—is frequently taught, but it is generally of so theoretical a nature that it is of little value to the average nurse in her daily life. I should like every nurse to get some practical lessons in the domestic side of sanitation—learning to understand and handle drain-pipes, to study the meaning of trapping, and to know from a sound common-sense plumbing point of view the danger of undisinfected discharges, and the simple science of avoiding drain-blocks. Nurses doubtless will say: "We cannot stand any addition to the studies already imposed on us. At present it is almost more than we can manage to get through our 'exams.' and cram-up in all we are expected to know."

But it is far from my philosophy to add on fresh subjects. Indeed, I would rather reduce the number of lectures given to nurses, and make these more practical and valuable. Recalling the generally useless nature of the hygienic lectures I attended when in hospital, I realise most keenly how much better it would have been to instruct us in every-day practical plumbing.

### The Dull Routine of Great Asylums.

THE VIEW OF AN ASYLUM VISITOR.

"A. B. A. L. H." WRITES: Though the treatment of modern times contrasts most favourably with that of the days of our grandfathers, there is still much to be desired. The subject of mental disease is in the abstract painful and repelling, but in view of the nervous and mental strain attending all arduous and sustained labour, and of the fact that, whether we know it or not, some of the world's best workers are inmates of asylums, we cannot ignore their sufferings without incurring the responsibility of helping to prolong them. There are men of intellectual culture, mechanical skill, and noble presence; and women whose best qualities are known only to their children and most intimate friends, suffering more or less keenly from conscious loss of liberty, from the sense of separation from their families, and, worse still, from doubt as to whether they will ever return to the sphere of life in which they formerly exerted an influence for good.

If hospitals for mental disease were more numerous, the following lines might never have been written. But having failed to obtain admission for a patient into either St. Luke's or Bethlehem, and the distance of St. Andrew's, Northampton, from London, being an insuperable disadvantage for one to whom frequent visits from home are essential, I know nothing of the advantages of those institutions, but have been told that in them recovery is distinctly aimed at, and in many cases is secured.

The editors of THE HOSPITAL "regard lunacy as a disease, and asylums as hospitals for its treatment." It would be well indeed if all concerned in the care and charge of so-called "lunatics" held the same views. The name asylum does not actually include the idea of healing: it is a refuge; literally "a place where one is safe from seizure." And from frequent visiting of patients in both private and public asylums, I cannot help thinking that the expectation and hope of effecting a cure in individual cases is too readily allowed to give place to a desire and conscientious effort to promote the general well-being of the multitude; the idea of giving "safety from seizure" to the patient is merged in that of giving to his relatives freedom from annoyance and responsibility, and to the public security from danger which *might* result from his vagaries. The medical staff of a public asylum seems differently constituted from that of a hospital, where we are accustomed to see names to which are appended the titles of house physician, house surgeon, and consulting physicians. The medical superintendents are indeed most able, clever, and humane, but the idea of cure is not as prominent as it might be. The honourable and highly-esteemed work of "nurse" in a hospital is here represented by that of attendant. But the proportion of attendants to patients in public asylums is so small, they can hardly be called mental nurses, and many have neither the qualifications nor the inclination to rise to the full height of their vocation. The public asylums are quite full, new ones are being erected, and additions are being made to many which already contain hundreds of patients. The increasing demand for accommodation makes it impossible to reserve rooms in the county asylums for private patients, who in former times were there received at a payment of £1 1s. per week, and were by that arrangement entirely separated from the pauper patients. The great size of the wards makes adequate classification most difficult, and patients of the middle class whose relatives cannot pay the terms of a private asylum have to occupy the same wards as the paupers. The payment of 9s. or 10s. per week required from those who can afford it makes no difference to the accommodation. The greatest sufferers, probably, by this condition of things are the wives and mothers of middle class male patients, who were the bread-winners of the family. What woman can endure the thought that her husband or son is in constant companionship day and night with men of a far lower position and entirely different habits from himself, many degraded before their affliction, some whose condition is the direct consequence of vice. If by an almost superhuman effort such a person is placed in a private asylum, many advantages may be gained, as the result of more individual attention, but still in these the proportion of recoveries is very small, and in many the very high terms are accounted for by luxurious appointments and amusements, which do not suit the tastes of a working man. There are many serious hindrances to the recovery of patients in public asylums, some of which require to be dealt with by Her Majesty's Commissioners. The congregating of one thousand or more patients in one asylum necessitates rules which are terribly irksome to a man who has been his own master—perhaps an employer of labour. What are they to the man of refinement and culture and literary tastes? There are clergymen in the county asylums. It is terrible to see the bent head and trembling walk of a man who has been in subjection to the discipline for many months. The indoor work connected with the general order and supply of daily wants is excessively monotonous. A visitor who spends the usual two hours with a patient, either in the visiting hall or in the ward, is painfully impressed with the continuous roar or rumble, reminding one of the noise of London traffic, and, at times, of the inside of a London railway terminus. It is explained when we see the stream of patients wheeling various burdens through the long corridors. The expression on their countenances seems to say, "You may come and you may go, but we go on for ever." The constant slamming-to of doors without handles, and the jingle of keys from a chain on the person of each attendant, have their own effect on us. How do these incessant noises affect nervous patients? The large number of patients renders individual companionship and conversation on the part of the attendants almost impossible, and tends to make them mechanical and arbitrary; they are, in fact, warders. And the difficulty of classification where the wards are so large makes some patients prisoners who, with more supervision, could have far more liberty. Among visitors to patients there can hardly be two opinions as to the great need of separate small wards, or the adoption of the "Cottage Hospital" plan. But the immediate practical object presented to readers of THE HOSPITAL is the benefit of individual interest in the patients. A frequent and regular visitor must observe the very small number of visitors (relations or friends) who come to see patients in public asylums. These become dull, lethargic, and hopeless from neglect and a sense of abandonment. And the suffering arising from want of sympathy is very great. Let it never be imagined that a "lunatic" or "insane" person is unconscious; that those who cannot control their actions are wholly unaware of the fact, that the man, who by some act of mania has lost liberty, home, and honourable employment, never yearns for the smile of those he loves, the laughter of little children, even the scent of the flowers he used to cultivate. Since every inmate of an asylum belongs to some parish, why are there no periodical visits from the parish workers? And as the mind is, next to the soul, the noblest part of man, why do we not find mental nursing taken up by men and women of general education, quick sympathy, power of conversation on thing natural and tangible to divert the harassed mind from things unreal and horrible. Give more freedom, more open-air labour, more sympathy from the outside world, more devotion on the part of attendants; and let the garden be the principal work-room, for in all life and growth there are suggestions of hope and expectation, and the world of nature contains secrets of healing never found in books alone.

⁂ Our correspondent has evidently kept her eyes open during her visit, and by taking advantage of her opportunities has been able to detect some of the undoubted evils of our present asylum system, especially the lack of individual attention to the extremely varying wants of the different patients, and the impossibility of arranging for that carefully graded classification which is so desirable for those who are just over the borderland of sanity. The difficulties, however, are very great. The mere matter of expense is no small affair; the constantly growing numbers of the insane who are every year thrown upon the rates, and the obvious economy of dealing with them on the large scale, making it very difficult indeed to introduce reforms in the directions indicated by our correspondent—reforms which are well recognised by many to be most desirable. For the demented, and for those whose mental life is passed absorbed in their own delusions, the matter is not of so much importance. But to those whose delusions are but intermittent, or affect only a small area of the mental field, the being massed with a crowd of dements, and being subjected to a discipline devised for the regulation of those who are liable to violent outbreaks and sudden impulses is a real evil, a form of punishment to which no one ought to be subjected if it can be avoided. The absence of proper accommodation for paying patients at so many asylums is a matter to which we have again and again drawn attention. Even where no payment is in question the compulsory companionship of people of different rank in life, of different tastes and different habits—a companionship which every day forces on them afresh the consciousness of their condition, and prevents them for a moment forgetting their captivity, is an influence which operates against recovery, while to those who pay it is an insult added to an injury, both of which they feel. The question is a wide one, and has many sides. Even whether recovery should be aimed at, or whether asylums should not rather be looked on as places of permanent segregation is a question which is open to a good deal of argument on either side. It is because the letter of our correspondent—who seems to think that all is so plain, and that the way of reform is so simple and straightforward—touches upon matters of finance, of treatment, of administration, and of sociology, that we insert it, with the hope of eliciting the views of those who have had a larger and more intimate acquaintance with the subject than she pretends to.—Ed. *T. H.*

### Where to Go.

Victoria Commemoration Club.—Dr. Maclean has resumed his most interesting lectures on "Gynæcological Operations" after the Easter recess. The next will be given on Tuesday, May 4th, at 3 p.m.

Grosvenor House.—A meeting on behalf of the Mary Wardell Convalescent Home for Scarlet Fever will be held at Grosvenor House, by permission of the Duke of Westminster, on Monday, May 17th, at 3.45 p.m.

National Society for Employment of Epileptics.—The Hon. Thos. F. Bayard will lay the foundation-stone of a new Home for Men at the Colony, Chalfont St. Peter's, Bucks, on Thursday, May 6th, at 3 p.m. At the same time Mrs. Bayard will open the Home for Women (Eleanor House). Train from Baker Street, 1.37 p.m., to Chorley Wood Station.

Trained Nurses' Club, 12, Buckingham Street, Strand, W.C.—A course of instruction in midwifery and monthly nursing will begin at the club on Monday, May 3rd, finishing in time for the July L.O.S. Examination in July. The courses of lectures may be taken separately if so desired. The next meeting of "Nurses in Council" will be on Friday, May 21st, at eight o'clock. Subject: "The Daily Nurse." A lecture on "Antipathies" will be given by Dr. Tom Robinson on May 28th, at eight o'clock.

### Cyclists and Saddles.

We have received a little pamphlet, entitled "Dangers of Cycling: A Warning to Ladies," which, if it is to be believed, is a warning with a vengeance! It appears that all along through the varied course of its evolution the bicycle has been constructed with an eye solely to the requirements of the "male creature." Its especial wickedness is the saddle, which renders cycling "physically and morally dangerous, and absolutely abhorrent to every rightly-constituted and self-respecting woman. The exercise becomes veritably a continuous act of public obscenity . . . a flaunting in the face of all the world that our modesty is dead, and an invitation to the man in the street to treat us accordingly. What should we think of a woman who permitted herself to be carried about in public astride a broom handle?"

Now this is all very fine; but is it quite true? We cannot help feeling that we have got hold of a veiled advertisement of a split saddle; but, in any case, we think that the statements contained in the pamphlet in question are gross exaggerations.

There is no doubt that a divided peakless saddle is a very good arrangement if fitted accurately; but then, if the ordinary saddle is arranged to fit the rider, it is quite certain that the bicycle can be used without the evils so forcibly insisted on by our author. What we are quite clear about is that if a female rider sits properly she ought not to allow the peak of the saddle to come near the pubic bone. Of course, if a lady bicyclist chooses to ride like a witch on a broomstick, then the saddle will "rise well up into the arch of the pelvis, and thoroughly bruise and grind against this bony roof the soft and extremely sensitive parts," &c. But then who does ride in that way? Such a seat would be impossible if the handles were in a proper position.

The narrow anterior part of the saddle, the so-called peak, is clearly not essential to a saddle—it is a mere question of the mechanical construction; but if it exists it is bound to be narrow to permit the full extension of the thighs. It is quite a mistake, however, to compare even the narrowest saddle to a broom handle. The line of suspension does not lie between the front and the back of the saddle, but between the front and the extreme outer edge at the part where the saddle is widest, but in any case the narrow part in front is not the part on which the rider sits. Whether a man or a woman, the rider should sit on the ischial tuberosities, and if this is done on a saddle of the right size the pubic bone and the parts attached are out of harm's way if the handles are the proper height and the rider sits properly. No saddles are made less in width than the distance between the ischial tuberosities, and if the wide portion is carried any further forwards than is necessary, it useless; nay, even injurious, for every extension of the thigh must tend either to cause abrasion of the adductor tendon or to throw the rider forwards on to the peak, the very thing to be avoided.

That the peak is an evil may well be admitted, whether in regard to men or women riders, and also that when riding carelessly a sudden jolt may bring it into contact with parts it should not touch. We cannot, however, admit for a moment that the ordinary saddle is responsible for such evils as are depicted by the writer of this terrible little pamphlet.

If a properly fitting saddle, even of the ordinary kind, is properly used we do not feel inclined to admit that it "is a crime against modesty and morality." The point of real importance in regard to any saddle is that it should fit. The machine and the saddle should be chosen separately; the one to suit the length of limb, the other to fit the sitting part, and that is not a matter depending altogether upon the shape of the pelvic bones, for the muscular development and the amount of fat have almost as much to do as the bony skeleton with the sort of saddle to be used.

### A Book and its Story.

"FOUR ENGLISH CATHEDRALS."

WHAT an endless fund of historical data, ecclesiastical and national, is to be found in the various churches scattered through the land. We are sensibly alive to the value of our cathedrals from an æsthetic point of view, but how few of us fully realise what a wealth of traditional significance is embodied in the sacred structures. In several cases they are diaries in the life of a nation, if one cares to read them so, for the spirit of a people is nowhere more evinced than in her houses of worship.

Quite recently four daintily illustrated little volumes * have appeared on this subject dealing with the histories of Westminster Abbey, Winchester Cathedral, York Minster, and St. Albans Abbey, to which the attention of the reading public should be directed. These small books supply a want long felt, and, if we are not very much mistaken, will only have to be known to be appreciated very highly.

"Westminster Abbey," written by Dean Farrar, comes from the pen of one who loved and venerated it, and with which the Dean's name will ever be associated. And that the writer fully appreciates the colossal proportions of his task is very evident from his introductory remarks on the subject of a single visit to the Abbey. "What such a visit might be to a man of universal knowledge, unlimited interest, and complete sympathy, no one can understand; for no single person possesses or can possess the consummate culture which would be requisite for the reception of such full impressions."

First among the many succeeding matters of interest, the Dean points out the religious symbolism of the building. The structure is not accidental. Down to the minutest particulars it is "a theology in stone." The prevalent number is three—triple height, triple length, triple breadth—to remind us of the doctrine of the Trinity. "Its other predominant numbers are four, the numbers of earthly perfectness, the signature of the world and of Divine revelation; and seven, the signature of the Covenant, and of the seven spirits of God, and of the seven pillars of the House of Wisdom. Its structure is cruciform to remind us of the Atonement." Speaking of the parish church of St. Margaret's, in curiously close proximity to the greater building, Dr. Farrar tells us that it claims priority in point of age. The church was built as early as the days of the Confessor for the worship of the population. The Abbey was not intended for parochial service. The choir was the daily chapel of the Benedictine monks; for great national and ecclesiastical processions the nave alone was set apart. Dr. Farrar deals with the entire history of his subject, which alas, from limits of space, it is not possible to enter into more fully here.

Winchester Cathedral, with its monastery, Canon Benham tells us, was founded by Alfred, under the learned St. Grimbald, for the purpose of the education of the priests and young nobles of his Court. Within this "new" Minster, evolved out of an already existant building, King Alfred himself was eventually buried. At the time of the invasion of the Danes Winchester Cathedral witnessed constant warfare. The actual safety of the building was protected by various monster walls. Canterbury Cathedral, not so guarded, was burnt; fortunately, Winchester was spared. The remains of the protecting wall built by King Ethelwulf to defend the latter are still to be seen.

During the Middle Ages the city was much frequented by monarchs, two royal marriages being celebrated in the Cathedral. The royal dignity of Winchester waned considerably, as that of Westminster arose. "But no see," writes its present historian, "can show so many bishops who were canonised; none has had so many bishops who were also Lord Chancellors. There were eleven of them, the last being Wolsey." Canon Benham's description is richly interspersed with delightful illustrations of the Cathedral and quaint bits inside and outside it.

"York Minster," writes Dean Pusey-Cust, echoing a widely-expressed verdict, "is a 'thing of beauty,' in spite of ruthless improvements and fanatical zeal and Puritan Philistinism, and indiscriminating utilitarianism and ignorant restorations. In spite of these, and in consequence of these, perhaps, York Minster is what it is, and if we cannot recall all that tradition tells us once adorned its courts and enriched its sanctuaries, we can admire and appreciate what has come into our hands, and thank God that it is our privilege to worship in a house so worthy of His holy name." The history of the Minster is sketched out with the some care and accuracy which distinguishes the other little books of this companion series. The glorious early transepts and tower are attributed to Walter de Gray, King John's friend, For beauty of design and execution they are, perhaps, unrivalled on the continent. How much Archbishop Walter de Gray expended on the erection of the transepts is not now known. "I only know," the Dean remarks, in reference to this, "that the south transept cost £23,000 to restore fifteen years ago." York Minster had many rich benefactors. Four prelates in succession each during his tenure of office strove to promote the completion of the grand design. "No effort was spared, no personal self-denial evaded; clergy and laity alike shared in the enthusiasm of the moment, the Plantagenet kings, for the most part resident in York, by offerings and by influence encouraging and stimulating the good work." York Minster has lived through its good as its evil times, that rifling fingers and devastating fires destroyed much that had dignified the sacred building, "but," the Dean says, "enough survives to gladden eye and heart with the noblest evidences of mediæval work and taste, and tokens on every side abound to testify that, in these latter days, Yorkshiremen have been as ready to repair the decay of age, restore ravages, and support the glory of God's House as ever they were in days gone by."

The history of St. Albans Abbey has been entrusted to the Rev. Edward Liddell, to whom we owe an interesting account of this fine old building, and are indebted to him for a great deal of information of which we must plead a previous ignorance. The Abbey, as we all are aware, is of extreme antiquity. The traditional site of the martyrdom of St. Alban is marked in the north transept, and here, a few years after his death in 303, the first church was built, which, in the course of time, was succeeded by one of extended dimensions. As it now stands, three distinct styles of architecture are traceable in the Abbey—Norman, Early English, and the Decorated; and Eastern and Western Christianity are typified in the Norman architecture and in the pointed Gothic. The essence of the Eastern faith being, as Mr. Liddell points out, "Repose and quiet, unbroken custom and tradition; its symbols are the soft rounded arch, the prevailing horizontal lines. The essence of the Western faith is constant growth and endless aspiration, a spirit reflected in soaring arches and towering spires of the Gothic builders." We are unable here to give but the most cursory glance through this fascinating little volume, of which the writer in modest terms describes as a slight sketch which "by no means exhausts the interest of the cathedral church, for if the Queen of Sheba came to life and read this account, and were then to pay a visit to the Abbey of St. Albans, she would say once more, as she said of the glory of Solomon, that 'the half had not been told her.'"

* "Westminister Abbey," "Winchester Cathedral," "York Minster." "St. Alban's Abbey." (Isbister and Co., London, 1897. In separate form, illustrated, 1s. each.)

### Everybody's Opinion.

[Correspondence on all subjects is invited, but we cannot in any way be responsible for the opinions expressed by our correspondents. No communication can be entertained if the name and address of the correspondent is not given, or unless one side of the paper only is written on.]

### THE ABUSE OF UNIFORM.

"E. F. G." writes: Having seen in one of the daily papers lately letters referring to the adoption of hospital nurses' uniform by those who are not nurses at all, I should like to add my protest to this monstrous fraud for the purpose of giving it greater publicity, and with the view of suggesting some protection for those who are so grossly misrepresented. I know of a town in the South of England where it was the common practice of a certain class of women to masquerade the streets attired in the uniform of one of the hospitals there, and whose bad conduct and fast manners brought down much unmerited abuse upon the hospital in question. Could not some special badge be adopted, bearing a registered seal and worn outside the cloak, only procurable by those who can show their credentials? Surely this would be a worthy way of commemorating Her Majesty's long reign, and would appeal to H.R.H. the Prince of Wales, whose personal sympathy has been called out in such a practical manner lately in the interests of our hospitals. I am aware that there is a badge worn by those nurses who belong to the Princess of Wales's Pension Fund, but this is invisible under the cloak, and it seems to me that the Prince of Wales might well be appealed to to give the protection of his Royal name to those who are nobly fighting in the cause which is so near his own heart. Might I suggest a clasp or medal, surmounted by the Royal arms, and the year 1897?

### DISPENSING.

Miss Blanche E. Thompson, Hospital for Women, Birmingham, writes: Will you allow me to say, in reference to "How to Learn Dispensing" that I think the time of six months is too short for the practical work. The Women's Hospital, Birmingham, was almost if not quite the first to employ ladies as dispensers, and this was over twenty years ago, and now most of the hospitals and dispensaries in Birmingham and the neighbourhood have ladies as dispensers, who have for the most part begun their career at the Women's Hospital Dispensary, or become pupils of ladies who were formerly there. This being the case I am anxious you should know it, as I often read in The Hospital inquiries *re* learning to dispense. There is great care needed in the choice of pupils, and I would urge that those who undertake to train women dispensers should, where possible, insist that the first thing to be done is to pass the Preliminary Examination of the Pharmaceutical Society. Besides having got over the first step this ensures a certain amount of knowledge of Latin, and also shows whether there be an aptitude for study. I have been at the above hospital over fourteen years, and have during that time had considerable experience in teaching dispensing. There are now women dispensers at the following hospitals and institutions in the Midlands: In Birmingham, at the Women's Hospital, the Ear and Throat Hospital, the Skin and Lock Hospital, the Orthopædic Hospital, and at the Provident Dispensaries at Hockley, Sherborne Road, Sutton Coldfield, and the General Dispensary, Coventry Road; at the Warneford Hospital and at the Dispensary, Leamington; the Women's Hospital, Wolverhampton; and at the West Bromwich Hospital, near Birmingham; also Bromsgrove. Besides those who are dispensers to private doctors.

### ASYLUM NURSES.

"Two Asylum Nurses" write: It is with much pleasure that we have read "One of the First Thousand's" letter in your issue of last week, and we desire to heartily thank her for her effort on our behalf. Indeed, we all greatly feel the want of a "Home of Rest and Nursing" for those engaged in asylum work, and wish to make an appeal, through your valuable and widely circulated paper, to the public. Even the smallest contributions will, we are sure, be thankfully received. It does seem very hard to us that hospital nurses should always receive so much sympathy from the public that apparently none is left for the poor asylum workers. Surely, if those engaged in the care of the body require Homes of Rest those engaged in the care of the mind stand even more in need of these homes, for all who have any knowledge of mental nursing must admit that it is far more trying both to the nerves and body generally than ordinary sick nursing. Do you not think that it would be only just to devote a small portion of the funds which are now being collected for the benefit of nurses to the establishment of a "Jubilee Home of Rest and Nursing" for asylum workers? We hope that you will be able to find space for the insertion of this letter, for we are sure that in writing as we have done we have only expressed the opinion of all engaged in asylum work. Dr. Shuttleworth, of Ancaster House, Richmond, is the honorary secretary of the Association of Asylum Workers, and to him should be sent all contributions towards the "Home of Rest and Nursing."

### Presentations.

On Tuesday, April 20th, Miss E. Stansbie, who has resigned the post of matron to the City Hospital, Birmingham, was presented by the hospital staff with a handsome clock, and by the health inspectors with a writing cabinet, as a token of their esteem and regard. Miss Stansbie has been connected with this hospital for the past fifteen years. She leaves to take up a similar position at Park Hill, Liverpool, and carries with her every good wish from all those with whom she has so long worked.

### Appointments.

### MATRONS.

Nurses' Home, Salisbury.—Miss Ethel Laurence has been appointed Lady Superintendent of the Salisbury Nurses' Home. Miss Laurence was trained at the Sussex County Hospital, Brighton, where she subsequently became sister of one of the surgical wards. For two years she held the post of matron at the Victoria Hospital, Lewes, and for the past three years has been engaged in private nursing.

Crewkerne Cottage Hospital. — Miss Mary Frances Cooper has been appointed Matron of this hospital. Miss Cooper received her training at the Stafford County Infirmary, subsequently holding appointments as charge-nurse at Burton-on-Trent and at Veterton Infirmary.

We regret that owing to indistinct writing the name of Miss Newill, the recently appointed Matron to the Jenny Lind Infirmary, Norwich, was incorrectly spelt in last week's announcement.

### Minor Appointments.

Jaffray Suburban Hospital, Birmingham.—Miss Ethel Godfrey has been appointed Sister at this hospital. Miss Godfrey received her training at the Royal Victoria Hospital, Bournemouth, where she subsequently held the post of staff nurse.

Fulwood Workhouse Infirmary.—Miss M. Back has been appointed Superintendent Nurse at this infirmary. Miss Back was trained at Sheffield Union Infirmary, where she remained as charge-nurse, subsequently working as charge-nurse under the M.A.B. at the Small-pox Ships, Dartford, and at Tooting Fever Hospital. Recently she has held the post of night superintendent nurse at the East Dulwich Workhouse.

### Wants and Workers.

Will any of our readers help us to collect a few pounds together to assist a trained nurse who has broken down in health towards a long rest? She has been offered a home for a year in the house of a relative in America, and half the necessary passage money. Her doctors say complete change and rest is her only chance of regaining her health, but her own people are quite unable to bear the whole expense of the journey and other incidental expenses. We want, therefore, to collect £7 to help her in this matter. "The Hospital" Convalescent Fund heads the list with £2, and our readers will be doing a real good if they will interest their friends in finding the remaining £5, which will enable Nurse K. to book her passage to America next month. Hers has been a weary struggle of many months against great suffering, and the proposed arrangement offers her the only chance of being able once more to return to work. Contributions will be gladly received by the Hon. Secretary, "The Hospital" Convalescent Fund, 28 & 29, Southampton Street, Strand, W.C.

Can any readers of The Hospital help "Sister," Cottage Hospital, Cummock, Ayrshire, N.B., to get a bath chair for a poor young man of 25, a collier, who has fractured his spine in the pit, and would be able to get out could a chair be procured for him. An old or second hand one would be most gratefully received.

### The Victoria Commemoration Club.

THE Secretary has asked us to insert the following notices and items of interest in connection with the club :—

On Tuesday next there will be a club dinner at half-past six ; price, 2s. 6d. Members wishing to reserve a table must write beforehand to the Secretary. Menu : Sole à le Colbert, tomatoes farcies, lamb and mint sauce, new potatoes, cauliflower, apple tart and cream, olives aux anchois, and coffee.

After this week cold luncheons will be started at the club, consisting of lamb and mint sauce or other meat, salad, biscuits and cheese. Price 1s. to members.

THE DOLLS FOR DISTRICT NURSES.

The applications for the dolls will be attended to as soon as possible.

FLOWERS FOR THE CLUB.

The Secretary has received a gift of lovely flowers for the club from a member in Essex, which were very acceptable and much appreciated.

### The Book World for Women and Nurses.

[We invite Correspondence, Criticism, Enquiries, and Notes on Books likely to interest Women and Nurses. Address, Editor, THE HOSPITAL (Nurses' Book World), 28 & 29, Southampton Street, Strand, London, W.C.]

PRACTICAL HINTS ON DISTRICT NURSING. By AMY HUGHES, Superintendent of Nurses, Bolton Union Workhouse, late Superintendent of the Central Training Home, Queen Victoria Jubilee Institute. (The Scientific Press, Limited, 28 and 29, Southampton Street, Strand. pp. 99. Price 1s.)

District nurses will find this handy little volume a mine of useful information. Many valuable hints are dispersed throughout its pages of a nature that show that they can only have emanated from a source at once practical and reliable. Miss Hughes rightly insists that if such work is to be continued on right principles, it must be entirely free from all pauperising influences. To ensure success the work must be done without suspicion of almsgiving. And this is not the least difficult of the many lessons a district nurse has to learn before she can be considered fully equipped for this branch of her profession. We are told that it is the opportunities given by district nursing that make it so important; the moral influences it affords, to which there is practically no limit. Habits of thrift may be induced, self-help, self-restraint, and self-respect encouraged. The impression which exists that highly-trained nursing is wasted in such work is an error due to ignorance, for the cases are many of them identical with those found in hospitals, with all the disadvantages of unfavourable surroundings thrown in. District nurses must understand all about the art of bandaging, they must also know how to nurse tracheotomy cases, hot air and vapour baths, hot and cold packs, dry cupping, leeching internally and externally, washing out of the bladder, hypodermic injections, the application of poultices and fomentations, and all the other details that are included in the education of a highly-trained nurse. There must also be courtesy, tact, and refinement, for, however poor he may be, "an Englishman's home is his castle," and he is morbidly sensitive when there is a tendency to ignore this principle. Much stress is laid on the uniform, which should be as light and convenient as possible. Veils are said to be a mistake for district nurses—a sentiment in which we fully concur, and, going further, would say for *any* nurse. Absolute neatness should be aimed at in appearance. There are several useful suggestions as to the hours of making the daily round, the time occupied in each case, what is required, and how it should be arranged. The remarks on district hygiene are excellent, for it is the most difficult of all subjects to impress on the poor, and the advice to nurses in infectious cases is clear and to the point, admitting of no misconception. The book concludes with some admirable remarks on maternity nursing, which alone are sufficient to ensure for it a well-deserved popularity.

### Notes and Queries.

*Army Nursing Reserve.*

(227) Please give me some information respecting the Army Nursing Reserve, terms and conditions of service, &c.? I have a form of application, but it does not give all the particulars I want to know.—*Enquirer.*

If you want more information than is given in the printed regulations to be obtained from the War Office, apply there for the further particulars you desire. Address the Adjutant-General of the Forces, War Office, Pall Mall, S.W.

*Training.*

(228) Can you tell me what the average height must be for a probationer, and whether having to wear glasses for reading would hinder one in getting into a London hospital for training? Will a knowledge of German be any good?—*Anxious.*

It is quite impossible to lay down rules respecting height, &c. Your acceptance at any hospital will depend on your general health and strength and fitness for the work rather than upon any question of inches. If you are otherwise in good health the mere fact of using glasses will not stand in your way. An acquaintance with languages is always "good," but a knowledge of cooking and of the use of broom and duster are of more value to the would-be probationer.

(229) Can you tell me if after a year's training in a provincial infirmary I could get into a London hospital for one or two years so as to finish my training? I want more experience, but not to have to begin from the beginning again and serve for three or four years. Can you tell me of any London or provincial hospital where I could enter for two years? —*Probationer.*

You will not get into any good general hospital with only one year's training except as probationer, and no London general hospital will take a non-paying probationer for less than a three years' engagement. If you can pay you can apply at any of the London hospitals, which nearly all take paying probationers at a premium of £1 1s. a week. Otherwise we should advise you not to grudge giving up three years for the sake of gaining a valuable certificate. Of provincial hospitals, among others, we believe a two years' course is to be had at the Royal South Hants Infirmary, Southampton, the Salisbury General Infirmary, or the Derbyshire General Infirmary. For future use it is far better to have a three years' certificate from a well-known institution than merely to possess the testimonials given for short periods to paying pupils.

*In Event of War.*

(230) In the possibility of war breaking out in the Transvaal and of nurses being required, to whom or to what association should a nurse wishing to go out apply? Would it be possible to volunteer provisionally without belonging to any army nursing society? Is there any charge made for answering in the "Notes and Queries" column?—(*Hedwig.*)

You had better write to Mrs. Piggott, hon. secretary of the Colonial Nursing Association, Imperial Institute, S.W. There is no fee for replying to queries in this column.

*Medico-Psychological Association.*

(231) Is it possible to prepare for the Medico-Psychological examination by correspondence? If so, how should I set about it?—(*Nurse M.*)

Write to the secretary of the association, 11, Chandos Street, W. You will find particulars about the examination and specimens of the questions set in "How to Become a Nurse" (Scientific Press, 28 & 29, Southampton Street, Strand, London, W.C.)

*Mental Training.*

(232) I am a trained nurse, and I want a short training in mental work in some good asylum. Please advise me.—*Nurse W.*

Write to Dr. Shuttleworth, Ancaster House, Richmond Hill, who is the hon. secretary of the Association of Asylum Workers.

*Soldiers and Sailors' Families' Association.*

(233) Please tell me what the above association is?—*Ireland.*

The Soldiers and Sailors' Families' Association was established in 1885 with the object of assisting the wives and families of men in the army, navy, and reserve forces. There is a branch for making grants to poor officers' widows, a clothing fund for supplying clothes to sick and invalided men returning from foreign service and to their families, and a nursing branch which supplies trained district nurses for soldiers and sailors' families in garrison and seaport towns. Secretary, G. W. Legg, 22, Queen Anne's Gate, Westminster.

*Lectures on Monthly Nursing.*

(234) Are there any lectures on monthly nursing given in connection with the lying-in hospitals which outsiders could attend?—*A Constant Reader.*

Write for information to the larger lying-in hospitals. There is a course on monthly nursing, beginning on May 3rd, at the Midwives' Institute, 12, Buckingham Street, Strand, W.C. Write for particulars to the secretary.

*Advice Wanted.*

(238) A young gentleman of good family, son of a clergyman, having served a four years' apprenticeship with a chemist, intends studying for the nursing profession. He requires, if possible, to be under the supervision of a medical man, and to be allowed to assist him in his dispensing and book-keeping, and at the same time to have time for study. Would the Editor of THE HOSPITAL favour him with advice in this matter through the medium of that invaluable weekly, THE HOSPITAL?—*Medicus.*

Our advice would be that the young gentleman should make up his mind whether he intends to be a chemist, a book-keeper, or a nurse. Nursing is not an art that can be attained by study. Actual training in a hospital is required, and while that is being undergone the question of being "under the supervision of a medical man" does not arise.

